# Salivary glucose in monitoring glycaemia in patients with type 1 diabetes mellitus: a systematic review

**DOI:** 10.1186/s40200-017-0287-5

**Published:** 2017-01-21

**Authors:** Cho Naing, Joon Wah Mak

**Affiliations:** Institute for Research, Development and Innovation (IRDI), International Medical University, Kuala Lumpur, 57000 Malaysia

**Keywords:** Diabetes mellitus, Diagnosis, Glucose, Saliva, Systematic review

## Abstract

**Background:**

Incidence of type 1 diabetes mellitus is increasing worldwide. Monitoring glycaemia is essential for control of diabetes mellitus. Conventional blood-based measurement of glucose requires venepuncture or needle prick, which is not free from pain and risk of infection. The non-invasiveness, ease and low-cost in collection made saliva an attractive alternative sample. The objective of this review was to systematically review the evidence on the relationship between salivary glucose level and blood glucose level in monitoring glycaemia in patients with type 1 diabetes mellitus.

**Methods:**

We searched studies which evaluate salivary glucose levels and serum glycaemia in type 1 diabetes mellitus in electronic databases of MEDLINE, EMBASE, Ovid and Google Scholar. We selected the eligible studies, following the inclusion criteria set for this review. Due to heterogeneity of studies, we conducted qualitative synthesis of studies.

**Results:**

Ten observational studies were included in this review, including a total of 321 cases and 323 controls with ages between 3 and 61 years and the majority were males (62%). Two studies were done exclusively on children below 17 years old. The significant difference between salivary glucose levels in type 1 diabetes mellitus and controls were reported in 6 studies with 8 data sets. Five studies with 7 datasets reported the correlation coefficient between salivary glucose and blood glucose in patients with diabetes.

**Conclusions:**

Findings suggest that salivary glucose concentrations may be helpful in monitoring glycaemia in type 1 diabetes mellitus. However, the utility of using salivary glucose level to monitor glycaemia should be evaluated in future well designed, prospective studies with adequate number of participants with type 1 diabetes mellitus.

**Electronic supplementary material:**

The online version of this article (doi:10.1186/s40200-017-0287-5) contains supplementary material, which is available to authorized users.

## Background

Diabetes mellitus, a group of metabolic diseases characterized by chronic hyperglycaemia resulting from defects in insulin secretion, insulin action, or both can be broadly categorized into type 1 diabetes mellitus (destruction of pancreatic beta cells, causing absolute deficiency of insulin), type 2 diabetes mellitus (a combination of decreased insulin secretion and decreased insulin sensitivity (i.e. insulin resistance) [1], gestational diabetes mellitus [2], other specific types of diabetes mellitus — monogenic diabetes mellitus/maturity onset diabetes of the young, genetic defects of beta-cell function or insulin action, diseases of pancreas, drug induced or associated with genetic syndromes and others (i.e. monogenic mutation in chromosome 12 or 7p, mitochondrial DNA, cystic fibrosis, hemochromatosis, endocrinopathies etc.) [1]. The incidence of type 1 diabetes mellitus is increasing worldwide both in low and high income populations [3]. Type 1 diabetes mellitus can affect people of any age, but usually occurs in children or young adults [1], the age group whose learning and earning potentials and capacities are crucial for the welfare of family and society at large.

Studies have documented that normalization of blood glucose levels can result in regression of diabetes associated complications such as peripheral neuropathy and peripheral vascular disease [4]. Repeated monitoring of plasma glucose in people with diabetes mellitus helps with timely identification of hyperglycaemia and is crucial for prevention of such devastating complications that can arise from poor control of the disease. The conventional method of blood-based investigations involve invasive procedures for blood sample collections from patients. This could evoke needle anxiety or a risk of blood-borne infections or both. Studies have shown that prevalence of needle anxiety in paediatric patients was 27% [5] and 22% of all patients of all ages in general practices [6]. Fear of sharp objects (needle) could further discourage some patients from monitoring their blood sugar levels regularly. It has been documented that 20.5% of those who reported needle anxiety avoided medical treatment involving needles [6].

Published studies have indicated the possible utility of saliva in monitoring of uric acid for cardiometabolic risk [7], drugs such as theophylline and steroids, quantitation of viral nucleic acids in herpes simplex virus DNA, varicella zoster virus DNA, and hepatitis C virus RNA [8], identification of biomarkers (IL-1β, -6, -8, TNF-α, lysozyme, MMP-8, TIMP-1) and total protein concentration in systemic diseases [9], *inter alia*. The non-invasiveness, ease and low-cost in collection have made saliva an attractive sample.

There has been a surge in published studies on the usefulness of saliva for monitoring of glycaemia. Some studies showed that salivary glucose levels were high in type 1 diabetes mellitus compared to controls [10] or were positively related to serum glucose in patients with diabetes, albeit with variation in the magnitude of relationships. Some studies reported differently [11, 12]. We are aware of the reviews looking at the usefulness of salivary glucose to monitor glycaemia in type 2 diabetes mellitus [13] or non-specified diabetes mellitus [14, 15]. Although all forms of diabetes mellitus are characterised by hyperglycaemia, their aetiology and pathogenesis vary. Acute, long-term and post-prandial hyperglycaemia are some of the conditions associated with endothelial dysfunction in type 1 diabetes mellitus [16]. Secretory activity of many exocrine glands (including salivary glands) depend on blood flow to these glands which in turn might depend on endothelial production of nitric oxide. Taken together, attention to the usefulness of saliva in monitoring of glycaemia in type 1 diabetes mellitus is valuable. The objective of the current review was to systematically review the evidence on the relationship between salivary glucose level and blood glucose level in monitoring glycaemia in patients with type 1 diabetes mellitus.

## Methods

We conducted this systematic review, following the guideline for the preferred reporting items of systematic review and meta-analysis (PRISMA) [17].

### Literature search

We searched studies which evaluate salivary glucose levels and serum glycaemia in type 1 diabetes mellitus in electronic databases of MEDLINE, EMBASE Ovid and Google Scholar. Search terms for studies were identified through medical subject headings (MeSH) as well as from those used for systematic reviews in similar context. MeSH terms and text words used were “saliva AND glucose” “sugar AND saliva” “saliva AND glycaemia” “diabetes AND saliva”, “saliva AND glycaemia”, “IDDM AND glycaemia” and “type 1 diabetes AND glycaemia”. The search was limited to publications in English language up to August 2016. We also manually checked the reference sections of the selected studies and relevant reviews for the possibility of any additional studies that might have been missed by the electronic search.

### Study selection

Studies were included if they were observational studies (survey, cross-sectional, case-control, cohort) carried out with minimum 10 participants of type 1 diabetes mellitus, regardless of age, gender and pregnant state and assessed correlation between salivary glucose concentration and blood sugar level in the same patient, compared mean salivary glucose levels between those with type 1 diabetes mellitus and healthy controls or those without diabetes mellitus.

Studies were excluded if they did not meet the inclusion criteria or assess participants with other comorbid diseases, which can alter salivary gland functions. Diabetes mellitus was confirmed (i) by self-report of patients with supportive medical records, (ii) as per the criteria by the expert committee on diagnosis and classification of diabetes mellitus [2], or (iii) by the American Diabetes Association (ADA) standards of medical care in diabetes [18].

### Data extraction from studies

One author (CN) screened titles and abstracts identified according to the selection criteria set for this review and this was cross-checked by the second assessor (JWM). The two authors then independently retrieved full-text copies of all articles that might be potentially relevant. The two authors then collected data from each study using a piloted spreadsheet. Data collected were author, year of publication, study country and setting, study design, original purposes, sample size, saliva collection, methods of glucose estimation and main outcome. The methodological quality of primary studies was assessed using the Newcastle-Ottawa Scale (NOS) [19]. The instrument used a star system to assess the study quality and the highest total score for a study that could be achieved was nine. During these processes, any discrepancy between the two authors was resolved by reaching a consensus by discussion.

### Statistical analyses

The studies identified for the current review used different outcome measures. As such, a variety of transformations were required to estimate the effect sizes [20, 21]. When studies reported ‘correlation coefficients’ between salivary glucose and blood glucose levels in persons with type 1 diabetes mellitus, we made transformation into approximate normality based on Fisher’s *z* score. If the correlation coefficient was not reported, we estimated Fisher’s *z* score from the *t* statistic of the linear association between salivary glucose and blood glucose. When studies reported the mean differences (± standard deviation, SD) of saliva glucose and blood glucose levels in type 1 diabetes mellitus and controls or non- diabetes mellitus, effect sizes for continuous measures were appropriate to apply. We considered Hedge’s standardized mean difference *g* as an effect size index described elsewhere [21–23]. Due to substantial heterogeneity of studies, we did not make the pooling of effect size estimates [24]. Statistical analysis was done with Stata 14 (TX: StataCorp LP). We reported the current systematic review, according to the PRISMA checklist [25] (Additional file 1).

## Results

**Fig. 1:**
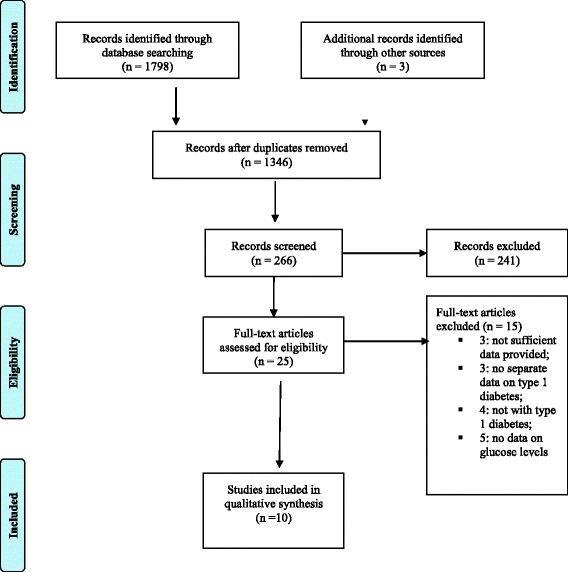
Study selection process

The four-phase study selection process is presented in Fig. 1. An initial search yielded 1708 citations. Of these, 25 studies were potentially relevant, and a final of 10 studies (with 12 data sets) [10, 12, 26–33] were included in the current review. The agreement between two investigators was substantial (kappa statistics 0.81).

The excluded studies were those which did not provide sufficient data on type 1 diabetes mellitus [34–36], did not give a separate data on type 1 diabetes mellitus [37–39], did not assess type 1 diabetes mellitus patients [38–41] or they did assess type 1 diabetes mellitus but not the glucose levels [42–46].

### Characteristics of the included studies

The main characteristics of the included studies are presented in Table 1. These studies included a total of 321 cases and 323 controls with ages between 3 and 61 years. Two studies were done exclusively on children with 17 years and below [10, 29]. Eight studies [10, 12, 27–31, 33] gave data on gender participants and the majority were males (62%, 158/255).

**Table 1:** Characteristics of the included studies

Study author, yr of publication [ref No]	country	type 1 DM	male/female	mean age of T1DM, yr	control(N)	fasting on collection	saliva collection	salivary glucose measurement	outcome (compared with controls)
Harrison, 1987 [26]	USA	30	NA	4–19	healthy controls (30) age, sex, race matched	1 h	stimulated	GOD	Salivary glucose, IgA, protein: significantly higher in T1DM More higher in uncontrolled T1DM
Darwazeh, 1991 [27]	UK	41 T1DM(17) & T2DM (24)	23/18	52 ± 16	non-diabetic (34)		unstimulated	enzymatic^b^	Salivary glucose: significantly higher in DM Salivary glucose & blood glucose concentration related.
Anderson, 1998 [28]	Sweden	Study A 10 (IGT: 10 (T2DM). Study B T1DM or T2DM (15); T2DM (9)	All males	Study A: 36–58; Study B: 32–76	healthy adults (24) 12 in A 12 in B.	10 h (overnight).	stimulated	enzymatic^b^	glucose in parotid saliva is elevated at least 2 h after glucose/ food intake in individuals with DM.
Belazi, 1998 [29]	Greece	10	5/5	4–15 years	healthy children (10) 5–17 years	2 h	unstimulated	GOD-POD	salivary flow rates: no difference salivary and serum glucose: significantly higher in T1DM.
Lopez, 2003 [10]	Argentina	20	11/9	3–15	controls (21) 5–12-years	8 h, except 2 h in 3 patients	unstimulated	GOD	salivary flow rate :diminished in DM Total sugars, glucose, urea, total proteins: greater in DM calcium values: decreased. Diabetic children have higher DMFT
Panchbhai, 2010 [30]	India	11 (Grp 1) + 8 (Grp 2) = 20	22/18 (Grp 1); 25/15 (Grp 2)	26–62 (Grp 1); 13–69 (Grp 2)	healthy non-DM (40)	2 h	unstimulated & stimulated	GOD	salivary glucose levels: significantly elevated in DM salivary amylase levels : significant decreases in DM
Vaziri,2010 [12]	Iran	40	19/11	9–61	healthy adults (40) T2DM(40)	overnight	unstimulated	Pars ^a^	Salivary IgA: no difference Salivary glucose: no difference Salivary flow rate: significantly lower in diabetic patients DMFT: higher in diabetic than controls
Nagalaxmi, 2011 [31]	India	50	28/22	7–20	controls (50), age & sex-matched	NA	unstimulated	GOPD	Significant positive correlation between salivary & serum glucose in T1DM
Behal, 2012 [32]	India	50 (T1DM& T2DM)	NA	57.8 ± 12 & 56.36 ± 11	non-DM (50)	NA	unstimulated	GOD	The mean level of salivary glucose: significantly higher in diabetes; A positive, but weak correlation between salivary and blood glucose
Shahbaz, 2014 [33]	India	30	16/14	9.7 ± 4.4 years	healthy controls (30)	overnight	unstimulated	GOPD	Salivary total protein: higher in T1DM Salivary flow rate: diminished in DM. Salivary albumin higher in T1DM. Salivary glucose levels: higher in T1DM. A highly significant positive correlation between serum & salivary glucose in T1DM.

*Alcohol* alcohol dependency, *chronic ds* disease, *h/o* history, *DM* diabetes mellitus, *DMFT* decayed, missing, filling teeth, *GOD* Glucose-oxidase method, *GOPD* Glucose oxidase- peroxidase method, *Grp* group, *IGT* impaired glucose tolerance test, *NA* not available, *Pg* pregnancy, *S* smoking, *yr* year

^a^: Pars method (glucose Oxidase Kit,Pars Azmoon Co, Tehran, Iran); ^b^: enzymatic ultraviolet detection method for glucose analysis (Boehringer Mannheim GmbH, Mannheim, West Germany, cat.No. 139041)

Four studies were done in India [30–33], and one each in Argentina [10], Greece [29], Iran [12], Sweden [28], the UK [27] and the USA [26]. Seven studies reported information related to blood glucose assessments. Four of these 7 studies (57%) obtained fasting blood samples [30–33] and measured glucose level with glucose oxidase-peroxidase method (GOPD) [27, 31–33]. For glucose estimations in saliva, four studies used glucose-oxidase method (GOD) [10, 26, 30, 32], three studies used GOPD [29, 31, 33], two studies used enzymatic ultraviolet detection method [27, 28], and the remaining one used kit-based GOD (Pars Azmoon Co, Tehran, Iran). The majority of studies (67%) collected saliva samples after at least 2 h of fasting [10, 12, 28–30, 33].

Six studies (8 data sets) reported the significant difference between salivary glucose levels in type 1 diabetes mellitus and controls [10, 12, 26, 29, 30, 33]. Five studies (7 data sets) reported the correlation coefficient between salivary glucose and blood glucose in patients with diabetes [27, 28, 30, 31, 33]. Three studies provided mean differences in two different groups of controls, healthy controls and control population with type 2 diabetes mellitus in one study [10] and poorly controlled/uncontrolled and well controlled/controlled diabetes mellitus in two studies [26, 30].

In general, the methodological quality of these studies were low due to poor quality in performance, reporting or both. Also, these studies failed to do (adequate) follow-up assessments for a stability of their findings. Five of the ten included studies (50%) in this current review could achieve 5 out of the maximum 9 stars given [10, 26, 27, 30, 31] (Additional file 2).

### Effect size

Two individual studies showed large effect size (Hedge’s *g* 3.75, 95%CI: 2.9–4.59) [26] and (standardised mean difference, Hedge’s *g* 4.27, 95%CI: 3.42–5.31) [33]. Due to heterogeneity of studies in view of variation in blood sample collection time and the saliva collection methods, pooling of standardised mean difference represented by Hedge’s *g* index was not attempted. The Harrison study [26] collected blood glucose 1 h after fasting, while the Shahbaz study [33] used overnight sample. Five studies with exclusively type 1 diabetes mellitus [10, 12, 26, 29, 33] reported the mean glucoses levels in saliva and blood and the effect size of each study in terms of Hedge *g* values were varied from a small effect size (*g* = -0.43) in the Vaziri study [12] to a substantially higher effect size (*g* = 4.37) in the Shahbaz study [33] (Table 2). Due to substantial heterogeneity of studies, the pooling of effect size was not done. A positive as well as large summary standard mean difference with non-zero overlapping in some of these studies [26, 29, 33] suggested there was some ground to believe that salivary glucose concentrations in the type 1 diabetes mellitus groups were higher than that in the controls.

**Table 2:** Distribution of the effect size of the difference between means salivary glucose levels in type 1 diabetes and controls

Study [ref]	Effect size, Hedge’s *g*	Lower 95%CI	Upper 95%CI
Shahbaz,2014 [33]	4.37	3.4	5.31
Behal, 2012 [32]	0.48	0.09	0.87
Panchbhai,2010^a^ [30]	1.31	0.82	1.8
Panchbhai,2010^b^ [30]	1.22	0.75	1.69
Vaziri,2010^a^ [12]	−0.03	−0.56	0.5
Vaziri,2010^b^ [12]	−0.43	−0.88	0.02
Lopez,2003 [10]	1.37	0.68	2.06
Harrison, 1987^a^ [26]	3.37	2.59	4.15
Harrison, 1987^b^ [26]	3.75	2.91	4.59
Belazi,1998 [29]	1.3	0.34	2.26

^a^:controlled or good controlled diabetes mellitus; ^b^: uncontrolled or poorly controlled diabetes mellitus

Five studies reported the correlation between salivary glucose and blood sugar levels in the type 1 diabetes mellitus groups. All these studies showed statistically significant correlations varied from weak positive (*r* = 0.11) to strong positive relationships (*r =* 0.99) (Table 3). Due to a substantial within study heterogeneity, it was not possible to make pooling of studies. Almost perfect relationship between blood and salivary glucose was found in two studies; *r* = 0.98 in one study [33] and *r* = 0.99 in another study [31].

**Table 3:** The relationship between blood and salivary glucose levels in type 1 diabetes

Study [Ref]	Sample size	Correlation coefficient	Z score
Andersson,1998 [28]	36	0.52	.5763397
Darwazeh, 1991 [27]	41	0.33	.3428283
Panchbhai, 2010^a^ [30]	40	0.4	.0400214
Panchbhai, 2010^b^ [30]	40	0.11	.1104469
Nagalaxmi, 2011 [31]	50	0.99	2.646653
Shahbaz, 2014 [33]	30	0.98	2.410142

^a^controlled diabetes mellitus; ^b^uncontrolled diabetes mellitus

Two studies [26, 30] provided data on those patients with controlled (well controlled) diabetes mellitus as well as those with uncontrolled (poorly controlled) diabetes mellitus. The Harrison study [26] showed that the mean salivary glucose level (mg/ml) was lower in the healthy controls (5 ± 1) or good controlled diabetes (11 ± 2) than the poorly controlled diabetes mellitus of 4–19 years patients (22 ± 7). The same pattern was found in a study by Panchbhai and associates (30); the mean salivary glucose level (mg/ml) was lower in the healthy adults (1.9 ± 1.4) than the controlled (7.69 ± 6.4) and uncontrolled adult diabetes mellitus (8.1 ± 6.5). As only two studies provided these information and the age group of participants in these studies varied, we did not make pooling of these studies.

## Discussion

Based on available data, the current meta-analysis provides insights to the utility of saliva in monitoring glycaemia in patients with type 1 diabetes mellitus. Findings suggests that salivary glucose concentrations in the type 1 diabetes mellitus groups were higher than in the controls. Due to a small number of studies with poor methodological quality and variation in reporting of outcome measures, there was no conclusive evidence on the usefulness of salivary glucose concentrations in monitoring glycaemia in type 1 diabetes mellitus.

Saliva contains serumnal components transported from blood capillaries into saliva by diffusion, active transport, and/or ultra- filtration via gingival sulcus. Hence, saliva can serve as a partial filtrate of blood for monitoring the health status of a person [47]. The rationale for the use of saliva in monitoring hyperglycaemia could have many explanations. Glucose is a small molecule which can easily diffuse through semipermeable membranes. The alteration in basement membrane of blood vessels (in patients with diabetes mellitus) leads to increased diffusion of glucose from blood to saliva [33]. Thus, large amounts of glucose concentrate in saliva of patients with diabetes mellitus [21, 26]. Moreover, salivary glands act as filters of blood glucose that would be altered by hormonal or neural regulations [10, 48] under several conditions of stress in humans [48]. Hence, salivary glucose level decreased after beginning insulin treatment in both children and adolescents [49], while infections (e.g candidiasis in those with dentures or HIV) and inflammation of salivary glands could increase the glucose level in saliva [31].

The substantial heterogeneity in the current meta-analysis could be due to a diversity in sample selection criteria (stimulated vs unstimulated, fasting hours prior to sample collection), methodological quality of study, underlying comorbid diseases as well as duration of diabetes mellitus and treatment status of diabetes mellitus (poorly controlled or well controlled). For instance, an individual study has shown a higher level of salivary glucose in the poorly controlled group than in the well-controlled group of type 1 diabetes mellitus (22.0 ± 7.0 μg/ml vs 11 ± 1 μg/ml) [26]. Thus far, we are not aware of any studies that provide sensitivity and specificity of salivary glucose levels in monitoring glycaemia in any type of diabetes mellitus. Future studies are required along this line of investigation.

There are some limitations in the present study. Salivary glucose concentration on glycaemia was time-dependent as the concentration of glucose in the parotid saliva remains elevated at least 2 h after glucose/food intake in both normal subjects and persons with diabetes mellitus [28, 37]. Hence the time and method of saliva collection can affect the estimated glucose level. Comorbid conditions could not be ruled out among the participants in the included studies. Many of the included studies were poor in the methodological quality and reliability of the findings is questionable. We did not perform age-specific analysis as there were large differences in the age groups of participants in these studies. This has important implications as the elderly groups are more likely to have other systemic diseases. Only two of ten studies in this meta-analysis were carried out in under 16-year-old children [10, 29]. An interpretation of our results should be aware of these potential confounding factors.

There may be confounding factors in the included studies such as the presence of co-morbidity, compliance to effective treatment, and age difference in participants. Inadequate data do not allow us to perform stratified analyses based on all these influencing factors. Due to the small number of studies with the small sample sizes, the possibility of type II statistical error cannot be ruled out, as the selected studies were not adequately powered to test for differences in the outcomes.

## Conclusions

Findings suggest that salivary glucose concentrations may be helpful in monitoring glycaemia in type 1 diabetes mellitus. However, the clinical utility of using salivary glucose level to monitor glycaemia should be evaluated in future well designed prospective studies.

## Additional files

Additional file 1: PRISMA checklist. (DOC 59 kb)
Additional file 2: Table S2. The methodological quality of the included studies. (DOC 53 kb)

## Abbreviations

ADA: American diabetes association
DM: Diabetes mellitus
GOD: Glucose-oxidase method
GOPD: Glucose oxidase- peroxidase method
IDF: International diabetes federation
NOS: Newcastle-Ottawa scale
PRISMA: Preferred reporting items of systematic review and meta-analysis
