# Supplementary material for: Salivary glucose in monitoring glycaemia in patients with type 1 diabetes mellitus: a systematic review
Source: J Diabetes Metab Disord. 2017 Jan 21;16:2. doi: 10.1186/s40200-017-0287-5 (PMC5251294; doi:10.1186/s40200-017-0287-5)
Supplement: Additional file 2: Table S2. — The methodological quality of the included studies. (DOC 53 kb) [file 40200_2017_287_MOESM2_ESM.doc]

**Additional Table 2** The methodological quality of the included studies

| 1^st^ Author | Selection | | | | Compar-ability | Outcome | | | | Total Stars |
| --- | --- | --- | --- | --- | --- | --- | --- | --- | --- | --- |
|  | representativeness | selection of non-exposed cohort | ascertainment of exposure | outcome of interested is not at the start of study comparability | comparability | (g) assessment of outcome (record linkage) | assessment of outcome  (independent or blind assessment) | (i) was follow-up long enough for outcomes to occur | adequacy of follow up of cohorts |  |
| Lopez, 2003 [10] | * | * | * | * | * |  |  |  |  | 5 |
| Vaziri, 2009 [12] | * | * |  |  | * |  |  |  |  | 3 |
| Harrison, 1987[26] | * | * | * |  | * | * | * | * |  | 6 |
| Darwazeh,1991  [27] | * | * | * | * | * |  |  |  |  | 5 |
| Anderson, 1998  [28] | * | * | * |  | * |  |  |  |  | 4 |
| Belazi, 1998  [29] | * | * |  | * | * |  |  |  |  | 4 |
| Panchbhai, 2010  [30]. | * | * | * | * | * |  |  |  |  | 5 |
| Nagalaxmi, 2011  [31] | * | * | * | * | * |  |  |  |  | 5 |
| Behal, 2012  [32] |  | * |  | * |  |  |  |  |  | 2 |
| Shahbaz, 2015.  [33] | * | * |  | * | * |  |  |  |  | 4 |
